# Extra Virgin Olive Oil (EVOO), a Mediterranean Diet Component, in the Management of Muscle Mass and Function Preservation

**DOI:** 10.3390/nu14173567

**Published:** 2022-08-30

**Authors:** Sara Salucci, Anna Bartoletti-Stella, Alberto Bavelloni, Beatrice Aramini, William L. Blalock, Francesco Fabbri, Ivan Vannini, Vittorio Sambri, Franco Stella, Irene Faenza

**Affiliations:** 1 Cellular Signalling Laboratory, Department of Biomedical and NeuroMotor Sciences (DIBINEM), University of Bologna, 40126 Bologna, Italy; 2 Department of Experimental, Diagnostic and Specialty Medicine (DIMES), University of Bologna, 40126 Bologna, Italy; 3 Laboratory of Experimental Oncology, IRCCS Istituto Ortopedico Rizzoli, 40136 Bologna, Italy; 4 Division of Thoracic Surgery, Department of Experimental, Diagnostic and Specialty Medicine-DIMES of the Alma Mater Studiorum, University of Bologna, G.B. Morgagni-L. Pierantoni Hospital, 47121 Forlì, Italy; 5 “Luigi Luca Cavalli-Sforza” Istituto di Genetica Molecolare-Consiglio Nazionale delle Ricerche (IGM-CNR), 40136 Bologna, Italy; 6 IRCCS, Istituto Ortopedico Rizzoli, 40136 Bologna, Italy; 7 Biosciences Laboratory, IRCCS Istituto Romagnolo per lo Studio dei Tumori (IRST) “Dino Amadori”, 47014 Meldola, Italy; 8 Unit of Microbiology, Greater Romagna Hub Laboratory, 47522 Pievesestina, Italy

**Keywords:** olive oil phenols, muscle mass loss, anabolic muscle pathways, sarcopenia, aging

## Abstract

Aging results in a progressive decline in skeletal muscle mass, strength and function, a condition known as sarcopenia. This pathological condition is due to multifactorial processes including physical inactivity, inflammation, oxidative stress, hormonal changes, and nutritional intake. Physical therapy remains the standard approach to treat sarcopenia, although some interventions based on dietary supplementation are in clinical development. In this context, thanks to its known anti-inflammatory and antioxidative properties, there is great interest in using extra virgin olive oil (EVOO) supplementation to promote muscle mass and health in sarcopenic patients. To date, the molecular mechanisms responsible for the pathological changes associated with sarcopenia remain undefined; however, a complete understanding of the signaling pathways that regulate skeletal muscle protein synthesis and their behavior during sarcopenia appears vital for defining how EVOO might attenuate muscle wasting during aging. This review highlights the main molecular players that control skeletal muscle mass, with particular regard to sarcopenia, and discusses, based on the more recent findings, the potential of EVOO in delaying/preventing loss of muscle mass and function, with the aim of stimulating further research to assess dietary supplementation with EVOO as an approach to prevent or delay sarcopenia in aging individuals.

## 1. Introduction: Skeletal Muscle Biology

Skeletal muscle mass homeostasis can be perturbated by aging, lifestyle-related causes such as a sedentary condition and reduced exercise or by severe disease. Skeletal muscle is a dynamic tissue with a crucial role in maintaining body metabolism and glucose homeostasis [1] thanks to its ability to react and rapidly adapt to external or environmental changes. Human health and survival are strictly dependent on skeletal muscle functionality as its loss increases the risk of falls, impairs mobility, and leads to muscle wasting, a condition correlated to cardiovascular disease, cancer, diabetes, cachexia, sarcopenia, and neurodegeneration [1].

Multiple molecular mechanisms are involved in the regulation of muscle mass and function. Muscle mass depends on a balance between protein synthesis and degradation [2]. It is well known that muscle hypertrophy occurs following an increase in protein synthesis, which can be induced by hormonal stimulation or resistance exercise. On the contrary, muscle atrophy typically results from reduced physical activity, neuronal alteration, or an increase in proteolysis. As regards proteolysis, two known proteolytic systems participate in the control of muscle size, the ubiquitin-proteasome system (UPS), which guarantees protein quality control, and the autophagy-lysosome system, which removes dysfunctional organelles and unfolded proteins [3].

**Figure 1:**
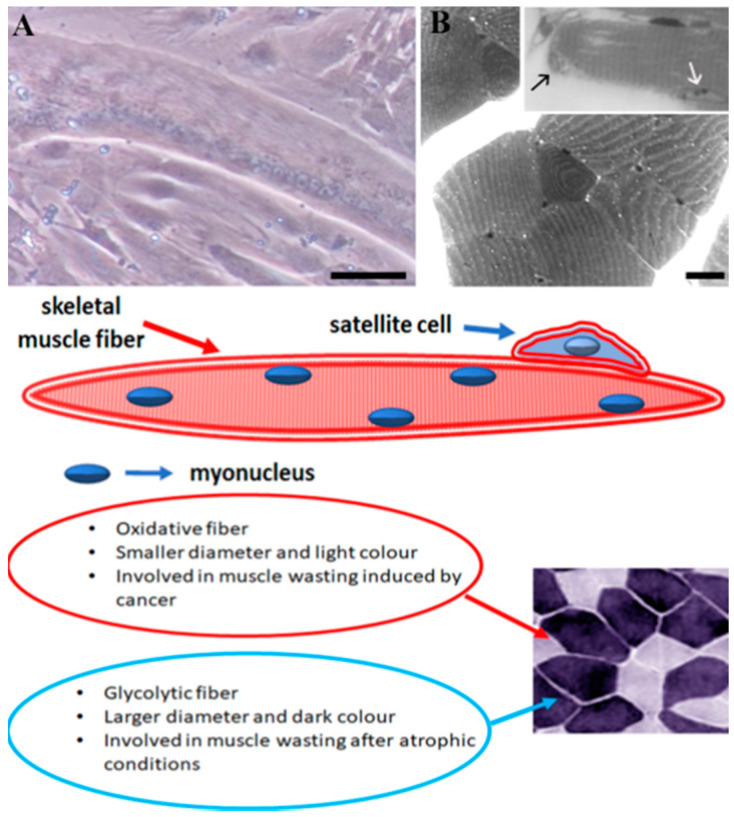
Image (**A**) shows a long and cylindrical adult myotube with several myonuclei located at the periphery of the fiber. Transverse (**B**) and longitudinal (inset B) optical sections of mouse muscle fibers where a satellite cell (black arrow) is located beneath the sarcolemma and basal lamina and a myonucleus (white arrow) is located at the periphery of the fiber in the space between myofibrils and sarcolemma. A schematic representation of a muscle fiber and inset photo where glycolytic and oxidative myofibers can be observed. Bars: 10 µm for A and 25 µm for B.

The complex modulation of skeletal muscle mass is strictly correlated with the anatomical characteristics of this tissue. Skeletal muscle mass is a syncytium with multinucleated and post-mitotic myofibers [4], which take origin from myoblast fusion to initially form multinucleated myotubes (Figure 1A). In adult skeletal muscle fibers, myonuclei, which are located between myofibrils and sarcolemma (Figure 1B, inset B and scheme), are post-mitotic and cannot divide. Therefore, myoblasts can either fuse with each other, forming new myofibers, or fuse, donating their nucleus, to an already existing myofiber [5]. Myofibers can be divided into fast (oxidative, intermediate metabolic properties) or slow (glycolytic, fatigue-resistant) fibers and respond specifically to a variety of stimuli, including hormonal levels, denervation, corticosteroids, aging, inactivity, and disease, as well as to metabolic and mechanical demands [6]. Fast fibers are affected by atrophic conditions, whereas muscle wasting induced by cancer affects slow fibers [7,8].

Tissue regeneration is a fundamental property of skeletal muscle, correlated with the activation and migration of a population of adult stem cells, called satellite cells, which proliferate and differentiate among the muscle fibers. Satellite cells, located between the basal lamina and sarcolemma (Figure 1, inset B and scheme), have a crucial role in multinucleated myofiber development, growth, and maintenance [9]. These cells, which are usually quiescent, become activated during myogenesis or during regeneration to repair damaged muscle [10]. Indeed, skeletal muscle development and regeneration also depends on satellite cell functionality, which is regulated by several molecular pathways [11] and by a family of known myogenic transcription factors [12,13,14,15,16]. Thus, a variety of anatomical components and molecular mechanisms participate to reach and maintain muscle mass homeostasis, a condition that can be perturbated by environmental stressors, inflammation, and oxidative stress, contributing to muscle wasting. In fact, muscle mass appears susceptible to inflammatory molecules leading to protein catabolism increase and consequent malnutrition [17]. Moreover, elevated reactive oxygen species (ROS) production, which correlates with muscle mitochondria alterations, induces post-translational modifications, which compromise muscle protein function in aged individuals [18]. Therefore, the identification of nutritional compounds able to interact with anabolic pathways to improve myofiber growth and differentiation, satellite cell function and intracellular organelle homeostasis represents an interesting issue within the field, with the aim of delaying the loss of muscle mass and function occurring in atrophic conditions. In this scenario, this review discusses the latest findings on the role of extra virgin olive oil (EVOO), a crucial component of the Mediterranean diet [19], in the preservation of muscle mass with particular regard to sarcopenia, a muscle-wasting disorder characterized by progressive loss of skeletal muscle mass, quality, and strength; all conditions which are associated with physiological aging [20].

## 2. Materials and Methods

This review initially (Section 3 and Section 4) describes the main molecular regulators and pathways involved in the control of protein synthesis and skeletal muscle mass and function, as well as sarcopenia development, by considering those articles published on the subject in the last twelve years. Articles considered were indexed in and retrieved from PubMed and/or Google Scholar using the following key words: skeletal muscle atrophy, muscle protein synthesis regulators, molecular pathways of muscle atrophy, sarcopenia, mechanisms of muscle loss and function, muscle loss and aging, mechanism of aging.

Later (Section 5 and Section 6), the relevance of EVOO to the nutrition field and its efficacy in counteracting the sarcopenic phenotype is discussed, considering both in vitro and in vivo studies carried-out in the last twelve years and retrieved from the same research motors, using the following key words: olive oil in the diet, beneficial effect of olive oil, olive oil and sarcopenia, EVOO and sarcopenia, EVOO and muscle loss, olive oil in preventing muscle mass, olive oil and muscle atrophy, Oleuropein and muscle mass, Hydroxytyrosol and skeletal muscle, Tyrosol and sarcopenia.

## 3. Protein Synthesis Regulators

**Figure 2:**
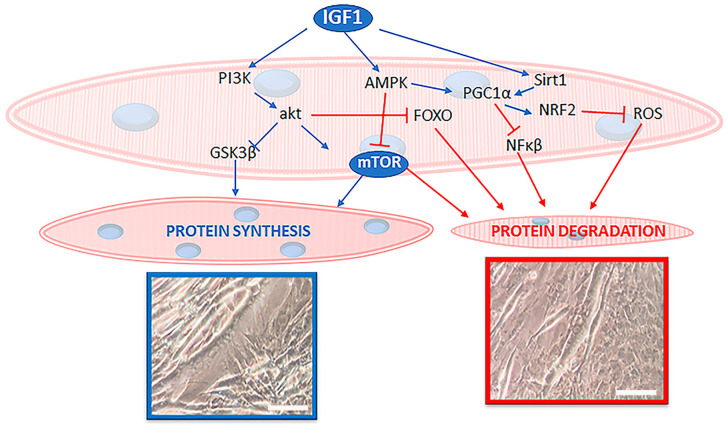
The scheme highlights the main anabolic actors, such as IGF-1 and mTOR, and their intracellular pathways in skeletal muscle biology. Signaling molecules that activate protein synthesis are colored in blue whereas those that inhibit protein synthesis and/or activate protein degradation are shown in red. Micrographs show hypertrophic (framed in blue) and atrophic (framed in red) cultured myotubes. Bars: 10 µm.

Insulin-like growth factor 1 (IGF-1), a key player in the regulation of glucose/energy metabolism, protein turnover and skeletal muscle function (Figure 2), is involved in the control of muscle growth, differentiation, and regeneration [21]. In young subjects, high circulating IGF-1 levels are positively associated with improved health and muscular endurance parameters. In contrast, high circulating levels of IGF-1 have a negative association with body fat, body mass index, and total serum cholesterol [22]. Low IGF-1 levels lead to chronic diseases, inflammation, and malnutrition [23]. Since it is the main influencer of both protein synthesis and degradation pathways in skeletal muscle, IGF-1 signaling is strictly involved in controlling myofiber size and function.

IGF-1 has two different isoforms, IGF-1Ea and IGF-1Eb. The differing roles of these isoforms remain unclear; however, IGF-1Ea appears to be the main isoform involved in satellite cell activation and growth, and its expression is tightly correlated with muscle hypertrophy; thus, it is fundamental for muscle mass maintenance during aging and in animals affected by muscular diseases [24,25,26]. IGF-1 controls protein synthesis by interacting with its receptor, IGF-1R, a receptor tyrosine kinase, to activate an intracellular signaling cascade that leads to the phosphorylation and activation of the phosphoinositide 3-kinase (PI3K)/AKT/mammalian target of rapamycin (mTOR) pathway. In this signaling cascade, AKT can phosphorylate and activate mTOR, thereby promoting protein synthesis [27]. At the same time, active AKT [28] leads to the inhibition of glycogen synthase kinase 3 (GSK-3), a metabolic kinase whose aberrant activity has been linked to inflammatory-mediated muscle decay, by phosphorylating GSK-3 on Ser21/Ser9 (-α/-β) [29]. The GSK-3β isoform, which is more expressed in skeletal muscle than the α-isoform [30], is considered a negative regulator of protein synthesis, and its ablation seems to favor atrophied skeletal muscle regeneration [29]. In fact, since active GSK-3β stimulates atrogin-1 and MuRF1 expression, two enzymes involved in UPS-mediated protein breakdown [31,32], it is not surprising that the lack or loss of GSK-3β prevents muscle mass and myofibrillar loss during atrophic conditions. Similar to AKT, other kinases, such as cAMP-dependent protein kinase A (PKA), protein kinase C-γ (PKCγ), protein kinase D1 (PKD1), protein kinase G (PKG) or mitogen-activated protein kinase-activated protein (MAPKAP) kinase-1, also mediate GSK-3β inactivation via Ser9 phosphorylation [33,34]. In addition, IGF-1 regulates protein synthesis by modulating the levels of myostatin, a member of the transforming growth factor-β (TGF-β) family that is secreted by skeletal muscle [35]. Elevated expression of myostatin down-regulates AKT, and this event is correlated with a reduction in myofiber size during aging as well as pathological conditions, such as cancer and cachexia [26].

As previously stated, IGF-1 plays a role in controlling protein breakdown, mediated by the UPS, via inactivation of GSK-3β. The UPS acts through two main E3 ubiquitin ligases, Muscle atrophy F-box (MAFbx)/Atrogin-1 and muscle RING finger 1 (MuRF1). MAFbx/Atrogin-1 and MuRF1 appear upregulated during disuse, denervation, inflammation, aging, glucocorticoid increase, and chronic diseases such as cancer, congestive heart failure, chronic kidney disease, chronic obstructive pulmonary disease (COPD), and AIDS [26,36]. In addition to GSK-3β, it is known that the IGF-1/PI3K/AKT pathway modulates both FoxO and NF-κB signaling, which are also known to regulate MAFbx/Atrogin-1 and MuRF1 expression. Therefore, both IGF-1 and AKT activation can inhibit muscle atrophy induced by inflammatory cytokines by acting on NF-κΒ expression [26]. Additionally, IGF-1 inhibits autophagic processes by the consequent inhibition of two pathways, unc51-like kinase-1 (ULK1) and FoxO3, which are involved in the induction of autophagy-related genes [37,38]. In contrast, IGF-1 can promote autophagy pathways with the aim of removing dysfunctional mitochondria that are responsible for excessive increases in ROS and muscle degeneration occurring during aging [21].

Moreover, IGF-1 is also involved in muscle function preservation through peroxisome proliferator-activated receptor gamma coactivator 1-alpha (PGC1-α) activation, an antioxidant marker that stimulates antioxidant defenses and promotes the maintenance of neuromuscular junction integrity, which are essential for muscle functionality. IGF-1-mediated stimulation of PGC1-α expression, also exerts a fundamental role in the control of mitochondrial dynamics by promoting the fusion and fission of mitochondria and by regulating their quality and functionality [39]. In this scenario, IGF-1 is supported by AMPK-dependent signaling, which guarantees the whole-body energy balance through the control of both glucose and lipid metabolism [40]. This collaboration between IGF-1 and AMPK pathways assures and improves mitochondrial biogenesis and appears to be strictly related to PGC1-α [41]. In addition, Sirt-1, a protein target involved in growth regulation, stress response, endocrine signaling, and extended lifespan appears modulated by IGF-1. Sirt-1 and AMPK comprise the main regulators of PGC1-α [21,42].

Satellite cell functionality is tightly controlled by intrinsic signaling pathways and extrinsic signals from the stem cell niche and also by circulating factors such as growth factors and hormones, including IGF-1 [43]. In fact, a lack of IGF-1-mediated pathway activation results in reduced expression of myogenic regulatory factors such as MyoD, Myf-5, and myogenin, with a consequent satellite cell function reduction [44,45,46]. Therefore, IGF-1 plays an important role in muscle homeostasis and preservation, leading to a reduction in muscle degeneration and inflammation while promoting the proliferation capacity of muscle satellite cells [47,48].

Another widely recognized regulator controlling muscle mass is mTOR, a serine/threonine kinase activated by various environmental and intracellular changes correlated with growth, including nutrient availability, hormonal stimulation, and energy status (Figure 2). mTOR functions as two distinct complexes [49]:

- mTORC1 (Raptor-containing complex) controls protein synthesis and organelle biogenesis by activating S6 kinase 1 (S6K1) and leading to the subsequent phosphorylation and sequestration of 4E-binding protein 1 (4EBP1), an inhibitor of the eukaryotic translation initiation factor 4E (eIF4E) [50]. Raptor deficiency leads to reduced post-natal growth, progressive dystrophy, impaired oxidative capacity, and increased glycogen stores. Moreover, mTORC1 inhibition blocks muscle hypertrophy in post-natal development and muscle regeneration [51]. For instance, in Pompe disease (a severe muscle wasting condition characterized by excessive accumulation of lysosomal glycogen the downregulation of mTOR) leads to a rapid progressive and lethal myopathy caused by a growth impairment [52]. It has also been demonstrated that the direct activation of mTORC1 stimulates protein synthesis and delays skeletal muscle atrophy induced by immobilization [53,54]. Additionally, activation of the PI3K/AKT axis by IGF-1 is sufficient to activate mTORC1 signaling, thereby inducing skeletal muscle hypertrophy [55]. Acute reactivation of AKT–mTORC1 also appears sufficient to counteract cancer-related muscle wasting, as demonstrated by Geremia and co-workers in a mouse model in which AKT could be selectively activated specifically in skeletal muscle [56]. In vitro studies on muscle cells showed that protein intake, as well as natural compounds (i.e., Tangshenoside I, Maslinic acid, Leucine) with antioxidant and anti-inflammatory properties, rescued muscle mass loss, induced by atrophic drugs, through the activation of PI3K/AKT/mTORC1 pathway and the suppression of catabolic signaling pathways [57,58,59]. In addition, Raptor loss, following AKT activation, was reported to reduce muscle hypertrophy and force, as well as mitochondrial protein content [60]. However, long-term continuous activation of mTORC1 appeared deleterious for skeletal muscle homeostasis, leading to dysfunctional autophagy and UPS activation [61].

- mTORC2 (Rictor-containing complex) is involved in AKT-dependent glucose and lipid homeostasis. The mTORC2 complex phosphorylates glucocorticoid-regulated kinase 1 (SGK1) to regulate ion transport and cell survival, protein kinase C (PKC) to modulate actin cytoskeleton organization, and finally AKT [62]. Activation of mTORC2 promotes embryonic myogenesis during development and the maintenance of muscle fiber homeostasis in adults. Its regulation appears crucial to satellite cell functionality [62,63].

Taken together, data collected on mTOR demonstrated its relevance in muscle growth, development, and survival. In this context, it is necessary to stress the point that several key cellular molecules act by reducing mTOR expression. For instance, AMPK activation can down-regulate the mTOR pathway. More specifically, under energy deficient conditions (AMP > ATP), AMPK phosphorylates mTOR, reducing mTOR signaling and, consequently, protein synthesis [64], a condition which leads to the development of an atrophic phenotype, including that of sarcopenia. It should also be mentioned that a number of cancer therapies showing the most promise in recent clinical trials target the PI3K/AKT/mTOR pathway [65]. The side-effects of these therapies on muscle homeostasis and quality of life will need to be closely monitored.

## 4. Muscle Mass Loss during Sarcopenia

Sarcopenia, defined as the age-associated decline in skeletal muscle mass and function, represents a well-established risk factor for most health-related conditions and events, including frailty, fractures, various disabilities, and death [66,67,68,69]. Muscle strength and muscle mass reduction are the two recognized components of sarcopenia [20] as defined by the 2010 European Working Group on Sarcopenia in Older People (EWGSOP), [66].

Skeletal muscle affected by sarcopenia shows severe alterations in cellular turnover and is characterized by abundant cellular vacuolization and mitochondrial damage, which compromise skeletal muscle homeostasis. Sarcopenia predominantly affects the type II (fast) muscle fibers with a size reduction of up to 50%, which is gradually replaced by type I fibers and fat-tissue deposits [70]. The loss of muscle mass is due to both muscle atrophy and myofiber death; conditions exacerbated by motor unit deterioration which finally results in loss of strength [71]. Several biological mechanisms have been proposed to explain sarcopenia development, including hormone imbalance (for instance IGF-1 deregulation), chronic activation of inflammatory pathways, and oxidative stress; in some conditions, such as myositis, there also appears to be involvement of an acquired immune response. All these conditions lead to mitochondrial dysfunctionality, altered proteostasis, aberration in muscle fiber composition, and reduced satellite cell potential. In particular, it has been documented that the loss of muscle mass and strength, that occurs during aging, is highly correlated with hormonal decreases, including IGF-1 and testosterone [71].

Growth hormone (GH, also known as Somatotropin), the main anabolic signal for muscle protein synthesis, appears impaired in sarcopenic subjects. As a consequence, lower serum levels of IGF-1, whose production is stimulated by GH, and functional performance levels have been observed in sarcopenic patients with respect to non-sarcopenic ones [72,73,74]. Furthermore, the IGF-1 impairment observed in sarcopenic patients is responsible for the up-regulation of myostatin and the associated deficiency in muscle differentiation, reduced protein synthesis, and enhanced protein degradation. The expression of IGF-1 also reflects the influence of inflammation, which accounts for a significant part of muscle mass loss occurring during aging [66]. In this context, it is known that low-grade chronic inflammation is involved in age-related diseases, including sarcopenia [75,76]. In fact, during aging, skeletal muscle cells produce inflammatory molecules able to induce losses in muscle mass, muscle strength and physical performance [77].

Several cytokines, such as tumor necrosis factor (TNF)-α, interleukin (IL)-6 and interferon-γ (IFN-γ), are implicated in the pathogenesis of muscle mass reduction associated with aging [78,79]. In particular, TNF-α is considered a potent trigger of muscle wasting in vitro and in vivo, through the inhibition of myogenesis and induction of apoptosis and proteolysis, via the activation of NF-κB and various UPS components [80]. Several authors documented high levels of TNF-α in individuals of advanced age compared to young individuals, and this finding appeared correlated with a reduced satellite cell number which contributed to sarcopenia development [81,82]. Furthermore, TNF-α up-regulation leads to inhibition of mTOR signaling and a reduction in muscle biosynthesis [83].

In addition to inflammatory cytokines, increased oxidative stress plays a crucial role in sarcopenia pathogenesis. During aging, mitochondria produce excessive levels of ROS and antioxidant defenses are less efficient to counteract this enhancement [84]. As a consequence, oxidative stress and lipid peroxidation events increase, leading to muscle fiber damage and death [85,86,87]. Strong experimental evidence indicates that the accumulation of mitochondrial dysfunction plays an important role in the muscle aging process, so much so that the progressive reduction in mitochondrial number and efficiency has been proposed as a mechanism capable of inducing sarcopenia [85,88,89]. Thus, age-related sarcopenia seems to be intimately linked to increased ROS production, increased mitochondrial apoptotic susceptibility, and reduced mitochondrial biogenesis.

The role of PGC1-α in the control of mitochondrial biogenesis appears crucial for skeletal muscle preservation. It has been demonstrated that a reduction in PGC1-α signaling leads to a decrease in AKT and mTOR expression. In contrast, in aged mice, it was observed that PGC1-α overexpression delays mitochondrial impairment, apoptosis, autophagy, proteasome activity, and muscle loss [90]. These findings highlight the significant contribution of healthy mitochondria to homeostasis and maintenance of muscle tissue, as mitochondrial changes can greatly contribute to age-associated muscle alterations [91,92,93].

During age-related sarcopenia development, an accumulation of dysfunctional organelles within skeletal myofibers represents a characteristic atrophic marker and favor impaired bioenergetics with consequent activation of aberrant catabolic pathways [94,95], leading to muscle wasting. Therefore, to delay sarcopenia development and progression during aging, it is necessary to identify compounds able to stimulate/activate anabolic pathways and to counteract pathways involved in muscle degeneration, such as those associated with inflammation and oxidative stress. Among these molecules, extra virgin olive oil (EVOO) seems to play a key role in modulating anabolic processes and in delaying muscle wasting.

## 5. Extra Virgin Olive Oil (EVOO)

**Figure 3:**
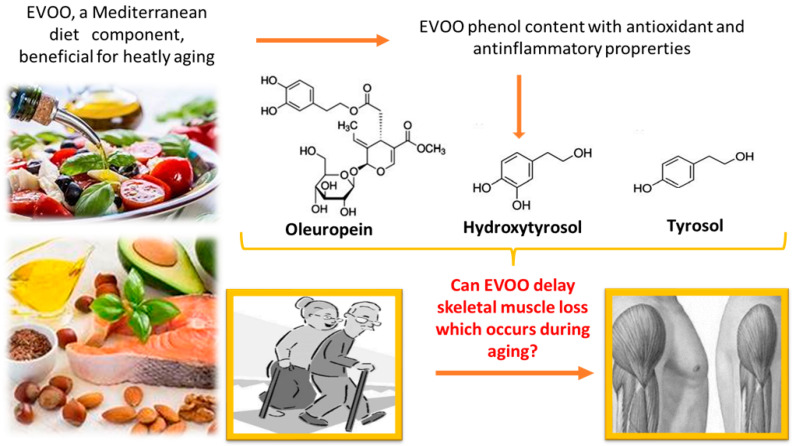
EVOO contains phenolic compounds such as Oleuropein, Hydroxytyrosol and Tyrosol with antioxidant and anti-inflammatory properties which could be useful for sarcopenia prevention.

EVOO, a central component of the Mediterranean diet, has an overall beneficial effect on human health. In particular, it appears to contribute to the prevention of metabolic disorders and cardiovascular disease [96,97]. It is known that consumption of olive oil has several advantages: (1) it reduces lipid and DNA oxidation, (2) it improves lipid profile and insulin-resistance, (3) it prevents endothelial dysfunction, (4) it has anti-inflammatory properties, and (5) it ameliorates blood pressure in hypertensive patients [97]. EVOO nutritional and antioxidant properties depend on the concentration of tocopherols, carotenoids, and phenolic compounds [98,99,100]. These latter can be divided into different classes such as phenyl ethyl alcohol (Hydroxytyrosol and Tyrosol), cinnamic (caffeic acid and p-coumaric acid) and benzoic (vanillic acid) acids, flavones (apigenin and luteolin), and secorroids (oleuropein and ligtroside derivatives). Phenolic compounds (Figure 3), in particular, Oleuropein, Hydroxytyrosol and Tyrosol, appear responsible for most beneficial properties attributed to EVOO by acting as potential scavengers of reactive oxygen species [101]. For instance, during aging they maintain genomic stability by protecting DNA (nuclear and mitochondrial) and cellular organelles (especially mitochondria) against oxidative stress and by stimulating endogenous antioxidant defenses [102]. Therefore, they are involved in delaying cellular senescence through the modulation of age-related chronic inflammation [103].

Two key modulators of human aging are integral parts of the inflammatory and oxidative stress responses: nuclear factor kappa-light-chain-enhancer of activated B cells (NF-κB) and NF-E2-related factor 2 (Nrf-2) [104]. NF-κB up-regulation characterizes several age-related and inflammatory diseases and is considered a hallmark of senescence [105]. In contrast, Nrf-2 levels appear to be down-regulated with age, as a result of epigenetic suppression or enhanced expression of its negative regulators [106]; as a consequence, cells and tissues are more vulnerable to oxidative stress, contributing to the age-related tissue degeneration. In this context, several studies have demonstrated that EVOO polyphenols protect cells and tissues against oxidative injuries and pro-inflammatory stimuli via promoting Nrf-2 signaling and by suppressing NF-κB activation [107]. Therefore, thanks to its phenolic content, EVOO shows a significant effect in modulating cellular pathways related to ROS and inflammation, and it appears interesting enough to further study its potential in preventing skeletal muscle wasting during sarcopenia.

## 6. Can EVOO Prevent Sarcopenia?

Reduction in muscle size and quality and an accumulation of fat deposits characterize the aging process in skeletal muscle. The excessive adiposity contributes to the physical decline that occurs during aging by promoting frailty, physical inactivity, and loss of independence, impairing the quality of life [108,109]. To date, there is no specific pharmacological treatment for preventing sarcopenia, only strategic interventions primarily focused on physical exercise and resistance training, which are able to partially restore muscle function in the elderly [110,111,112].

**Figure 4:**
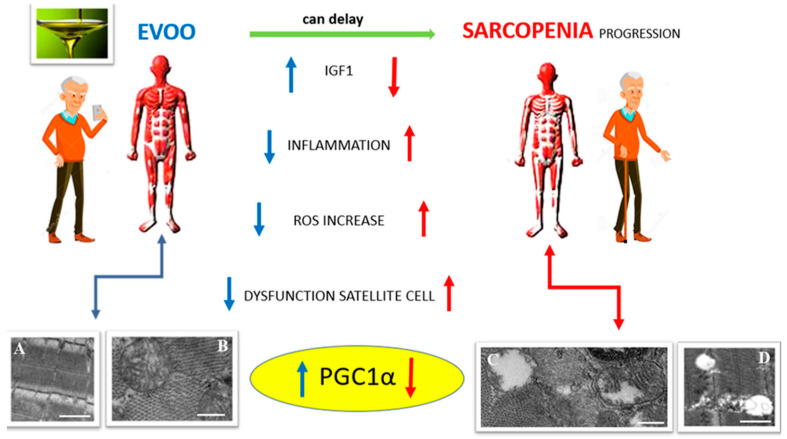
EVOO exerts a protective effect against sarcopenia. EVOO can upregulate IGF-1 expression, reduce inflammation and ROS, maintain satellite cell function and, in particular, acts by modulating PGC1-α expression, thus guaranteeing mitochondrial biogenesis and homeostasis. Electron micrographs of longitudinal (**A**,**D**) and transverse (**B**,**C**) sections of mouse skeletal muscle tissue show a preserved myofiber organization (**A**) and heathy mitochondria (**B**), or evident damage, with empty mitochondria (**C**), altered mitochondrial cristae (**C**) and degenerated sarcomere organization (**D**) following atrophic conditions. Bars: 1 µm for A and D; 250 nm for B and C.

In the last few years, researchers have been focusing their attention on dietary interventions as crucial tools to counteract sarcopenia; among these dietary interventions EVOO administration showed positive effects against aged-related muscle alterations. Even if the literature is not exhaustive regarding the role of EVOO in delaying the sarcopenic phenotype, some reports demonstrated that a regular consumption of EVOO has beneficial effects on body composition, including skeletal muscle (Figure 4), where an improvement of tissue morphology and function has been observed [113,114]. For instance, in older, obese subjects, EVOO administration during energy intake restriction stimulates protein synthesis and delays the loss of skeletal muscle mass and strength with an improvement in physical performance and quality of life [109]. Silveira and co-workers demonstrated that EVOO consumption associated with a healthy diet improved strength and muscle functionality in elderly, obese patients, highlighting its potential role in sarcopenia prevention [115].

González-Hedström et al., 2020 demonstrated that an oil mixture, composed of 75% EVOO and 25% algae oil, and administrated for 21 days favored a delay in muscle loss. In fact, aged rats treated with EVOO demonstrated higher gastrocnemius weight compared to untreated aged animals, and the decrease in protein content observed in the untreated aged rats appeared to be preserved in those treated with EVOO. In this experiment, EVOO counteracted muscle aging by reducing inflammation mediated by the inflammatory cytokine IL-6, modulated myogenin expression, and induced an increase in PGC1-α expression [116]. Moreover, these same authors also demonstrated the involvement of histone deacetylase 4 (HDAC-4) in sarcopenia development. Expression of HDAC4 was up-regulated in muscle obtained from aged rats and its elevated expression correlated with high levels of myogenin, which further activated a number of atrogenes. Treatment of aged rats with EVOO reduced expression of HDAC-4, leading to reduced skeletal muscle senescence [116].

As the beneficial outcome of EVOO in counteracting muscle loss depends on the phenolic compounds it contains, several studies have tested the individual activities of these compounds during atrophic conditions. For instance, Oleuropein, an EVOO phenol, demonstrated scavenger properties in C2C12 murine muscle cells, where it was able to counteract an excessive increase in oxidative stress [117]. In addition, it reduced mitochondrial oxygen species generation in primary-cultured chicken muscle cells through Sirt1 activation and PGC1-α expression, with a consequent reduction in oxidative potential and preservation of mitochondrial biogenesis [118]. Likewise, Hydroxytyrosol, a known antioxidant and the main component of the EVOO phenolic fraction, is derived from hydrolysis of oleuropein, and its concentration in EVOO depends on the altitude and latitude of the olive tree from which the olives and oil were harvested, the variety of olive, the collection time; and the processing conditions [119,120]. Hydroxytyrosol is able to scavenge ROS and to enhance endogenous antioxidant systems in several cell models, as well as to prevent alteration of mitochondrial dynamics, which plays a vital role during mitochondrial dysfunction-associated muscle disorders [120], including sarcopenia. Studies have also reported on the ability of Hydroxytyrosol to stimulate mitochondrial biogenesis, thereby protecting mitochondrial function, and inhibit apoptosis in strenuous exercise-induced skeletal muscle fatigue and in muscles of obese mice [121,122]. Wang and co-workers [123] demonstrated that Hydroxytyrosol could significantly prevent mitochondrial membrane potential and cell viability loss in myotubes exposed to high oxidative stress levels. These same authors showed that Hydroxytyrosol could also reduce excessive ROS by enhancing mitochondrial oxygen consumption capacity and activation of mitochondrial complex I and II [123]. Thanks to these properties, Hydroxytyrosol might be expected to have a beneficial role in counteracting aging, as well. In this regard, a positive outcome has been observed in skeletal muscle of aged rats treated for 6/8-weeks with a polyphenolic mixture containing Hydroxytyrosol, and low amounts of Tyrosol, catechol, gallic acid, homovanillic acid, and caffeic acid. This treatment improved the decline in skeletal muscle function attributable to aging-associated oxidative stress, restoring the resting cytosolic calcium concentration, sarcoplasmic reticulum calcium release, and preserving muscle weight and blood creatine kinase levels [124]. The protective effects of Hydroxytyrosol have also been observed in L6 skeletal muscle cells exposed in vitro to radical generator cumene hydroperoxide, a known pro-oxidant agent [125]. No data in the literature was retrieved regarding the effects of Tyrosol in skeletal muscle models or in connection with muscle aging, nevertheless Tyrosol is widely noted for its strong activity as a neuroprotective agent [126] and as an anti-inflammatory/antioxidant molecule [127,128,129]. Only a single paper, published in 2019, highlights the potential pharmacological application of Tyrosol in skeletal muscle tissue. In the reported study, Tyrosol was assayed as a potential small drug to treat therapeutic angiogenesis in diabetic patients affected by hindlimb ischemia. Tyrosol was reported to exert cytoprotective effects against hyperglycemia-induced oxidative stress in skeletal muscle cells, where it increased cell proliferation and acted by suppressing apoptotic death [130]. Thus, due to its many noted properties, Tyrosol deserves further detailed investigation in in vitro and in vivo models of skeletal muscle aging and sarcopenia. The Table 1 summarizes the data available on the effects of EVOO and its phenolic components in preventing skeletal muscle damage related to sarcopenia.

## 7. Conclusions

Data collected over the last ten years reveal EVOO, the main fat source in the Mediterranean diet, to be a dietary nutrient of considerable importance with regard to its potential benefits in maintaining skeletal muscle homeostasis during aging [131]. Increased incorporation of EVOO or its bioactive phenolic compounds into the diet could be a strategic intervention against age-related sarcopenia, a skeletal muscle disease associated with adverse outcomes due to a progressive loss in muscle mass and function as a consequence of a sedentary lifestyle and age-related metabolic changes. This review focuses on the potential usefulness of EVOO consumption to promote an increase in skeletal-muscle protein synthesis rates and stimulate an anabolic muscle response, thus allowing, at least in part, for an attenuation in muscle wasting and a delay in sarcopenia progression. The beneficial properties of EVOO are strictly related to the phenolic content, which represents a minor fraction of EVOO molecules. These compounds demonstrate a strong ability to activate anabolic pathways and to counteract age/disease-related changes involved in muscle degeneration, such as mitochondrial alterations and inflammatory processes [132]. In particular, several studies point to the role of EVOO in maintaining mitochondrial homeostasis through modulation of Sirt1 and PGC1-α expression (Figure 4), and this data appears extremely interesting, especially in light of the fact that accumulation of dysfunctional mitochondria is a major contributing factor to the development of sarcopenia [133,134,135]. Therefore, examining more closely the efficacy of EVOO phenols and studying their mechanisms of action in skeletal muscle models of aging both in vivo and in vitro are essential for designing new therapeutic approaches with the aim of treating sarcopenia. Such studies would also benefit from randomized controlled human trials to assess if EVOO addition to the diet in conjunction with standard interventive measures, such as resistance training and exercise, enhances muscle mass and function, and, above all, quality of life in individuals affected by or at risk of sarcopenia.

## Figures and Tables

**Table 1:** Human, animal and cell studies have been schematized to highlight the sample size (*n*), the dosage end the time of administration of EVOO, Oleuropein, Hydroxytyrosol and Tyrosol.

	Human Studies	Animal Studies	In Vitro Studies
**EVOO**	○ - Heathly adults (*n* = 45) 50 mL/d EVOO for 30 days ○ - Older obese subjects (≥60 years, *n* = 73), 40–60 mL/d EVOO for 12 weeks, isocaloric diet ○ - Obese subjects (18–64 years, *n* = 50), 52 mL/d EVOO for 12 weeks in DietBRa program	Old rats (*n* = 8), 2.5 mL/kg EVOO for 21 days	No data
**Oleuropein**	No data	No data	○ - C2C12 myotubes exposed to oxidative stress, pre-treated with 100–600 μM oleuropein for 24 h ○ - Primary cultured chicken muscle cells exposed to Oleuropein added to the culture medium at 0.1% volume (*v*/*v*)
**Hydroxytyrosol**	No data	○ - 4-week-old male C57BL/6 mice (*n* = 10), 10 mg/kg/day or 50 mg/Kg/day Hydroxytyrosol with a high-fat diet ○ - Sprague–Dawley (SD) male rats, 25 mg/kg/day Hydroxytyrosol, endurance exercise	L6 myotubes treated with 10 μL/mL or 50 μL/mL of Hydroxytyrosol
**Tyrosol**	No data	No data	C2C12 cells after hyperglicemia induction were treated with 50 mg/mL Tyrosol for 24 h
